# Supplementary figures and images for: The Toxoplasma Acto-MyoA Motor Complex Is Important but Not Essential for Gliding Motility and Host Cell Invasion
Source: PLoS One. 2014 Mar 14;9(3):e91819. doi: 10.1371/journal.pone.0091819 (PMC3954763; doi:10.1371/journal.pone.0091819)

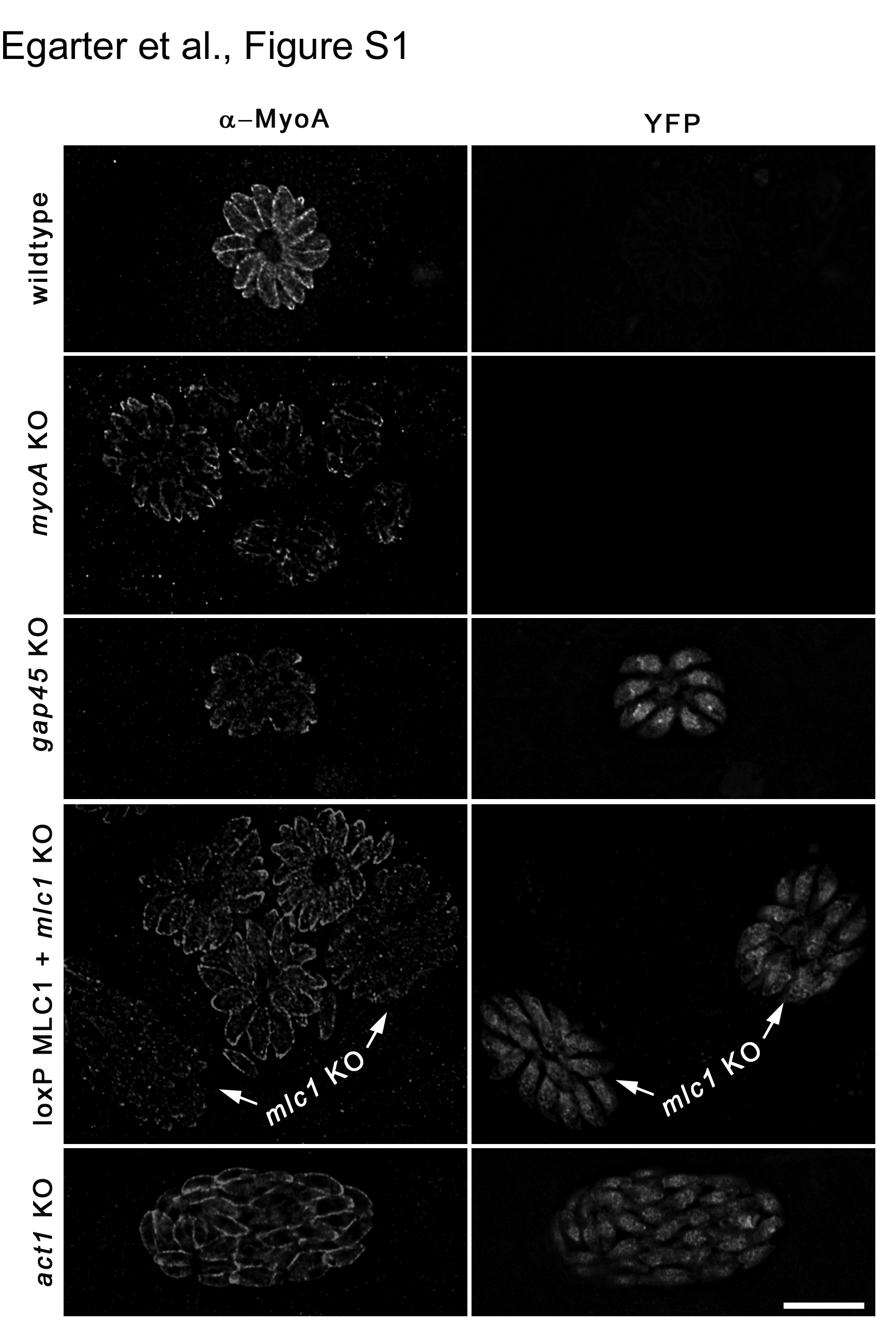

Supplement: Figure S1 — Comparison of MyoA antibody in various strains. Using the MyoA antibody for different strains revealed an unspecific background level within myoA KO parasites. Compared to wildtype parasites the signal was not detected at the periphery of the parasites and seemed mainly concentrated at the apical region of the parasites. Although no conclusion can be drawn concerning a possible depletion of MyoA in mlc1 KO parasites, a clear localisation change (periphery to cytosol) of MyoA within mlc1 KO and gap45 KO can be observed. Scale bar: 10 μm. (TIF) [file pone.0091819.s001.tif]

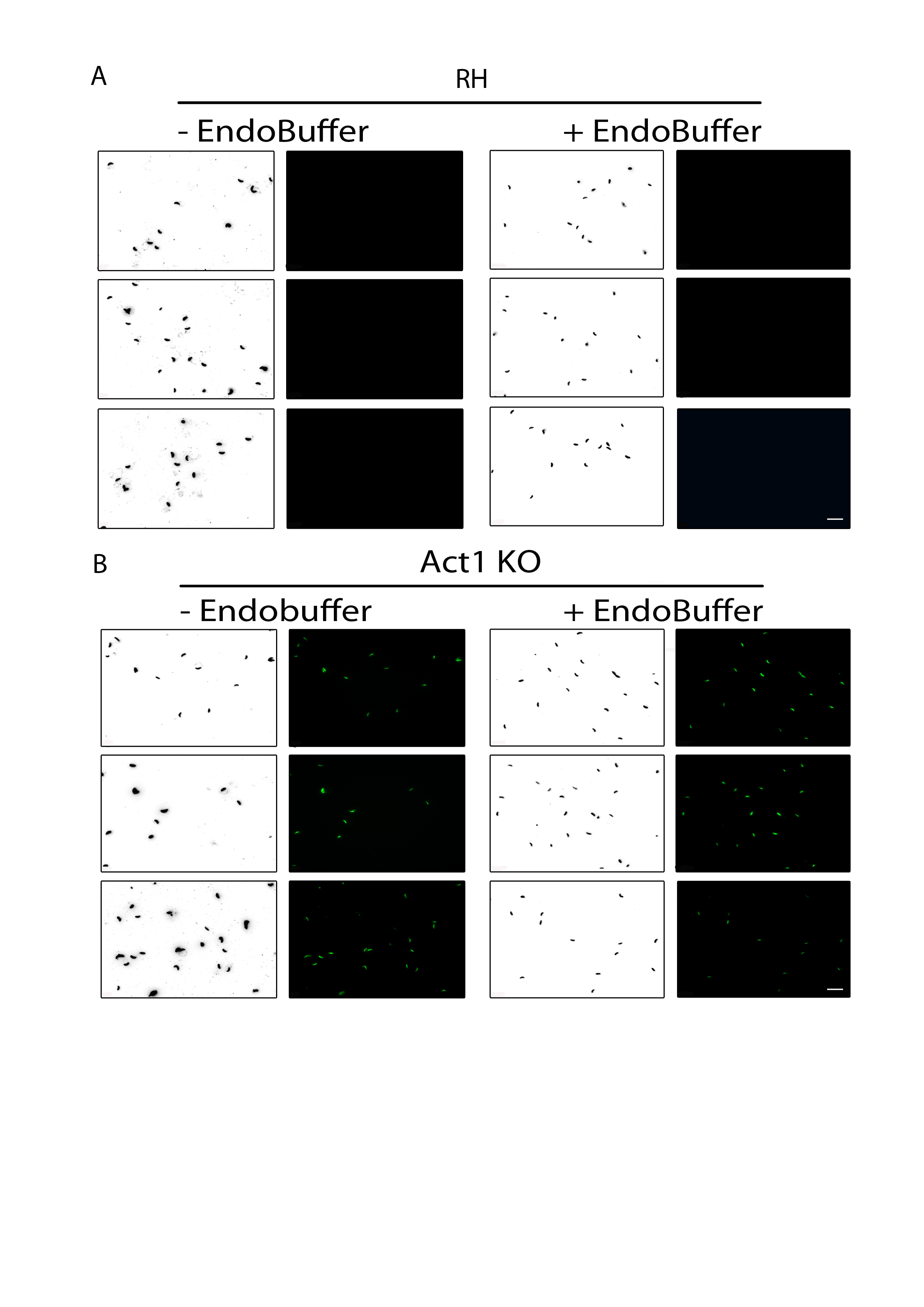

Supplement: Figure S2 — Gliding motility in absence of Act1. Trail deposition assay of RH Δhxgprt (A) and act1 KO (B) parasites. Parasites were allowed to glide either in gliding or endo buffer (high potassium buffer). Act1 KO parasites were analysed 96 hours post rapamycin induction (as indicative by green fluorescence). In both cases no gliding trails were observed in Endo Buffer. Scale bar: 20 μm. (TIF) [file pone.0091819.s002.tif]
