# Supplementary material for: The Toxoplasma Acto-MyoA Motor Complex Is Important but Not Essential for Gliding Motility and Host Cell Invasion
Source: PLoS One. 2014 Mar 14;9(3):e91819. doi: 10.1371/journal.pone.0091819 (PMC3954763; doi:10.1371/journal.pone.0091819)
Supplement: Table S1 — Summary of oligonucleotides used in this study. (PDF) [file pone.0091819.s003.pdf]

Table S1. Summary of oligonucleotides used in this study

Oligo	5'-3' sequence	final vector	parental line
**3′UTR MLC1 fw**	CGGAGCTCGTTGGATCGAAATTTTGGCCGGCATTTCTCG	*loxPMLC1loxP–YFP–HX*	ku80::diCre
**3′UTR MLC1 rv**	GGGAGCTCAATTATCGGTGCCATCTTCGCGTAACAAACGCGG	*loxPMLC1loxP–YFP–HX*	ku80::diCre
**MLC1 ORF fw**	GGGAATTCGACAAAATGAGCAAGGTCGAGAAGAAATGCCCGG	*loxPMLC1loxP–YFP–HX*	ku80::diCre
**MLC1 ORF rv**	CGTTAATTAATTACTCCCTTCGCTCGAGCATTGCC	*loxPMLC1loxP–YFP–HX*	ku80::diCre
**5′UTR MLC1 fw**	GCGGGCCCCCAAGCATTCATCTCCGCACAGCGGG	*loxPMLC1loxP–YFP–HX*	ku80::diCre
**5′UTR MLC1 rv**	CGCTTAAGTATAACTTCGTATAATGTATGCTATACGAAGTTATCTTGAGACCAGTTGGCAGGCGCAACGCGAAAGGCT	*loxPMLC1loxP–YFP–HX*	ku80::diCre
**GAP45 ORF fw**	GGGAATTCATGGGAAACGCGTGCAAGAAGAAC	*loxPGAP45loxP–YFP–HX*	diCre Δku80
**GAP45 ORF rv**	GGGTTAATTAATCAGTTCAACAAGGGTGCATCCGAC	*loxPGAP45loxP–YFP–HX*	diCre Δku80
**5’UTRGAP45 fw**	GCGGGGCCCCTTTAGCGTGCCAGAGGGTTTTGGC	*loxPGAP45loxP–YFP–HX*	diCre Δku80
**5’UTRGAP45 rv**	CGGAATTCTATAACTTCGTATAATGTATGCTATACGAAGTTATCGAATCGAAAAAGTGCGAAAAAGTTGAGGGGGCGAC	*loxPGAP45loxP–YFP–HX*	diCre Δku80
**3’UTR GAP45 fw**	CCACATGTGCTTCGGGATCTGTGTCTGTATAGCGTGC	*loxPGAP45loxP–YFP–HX*	diCre Δku80
**3’UTR GAP45rv**	GCGCCACATGTCTCATCTGTCATAGTTCCTGG	*loxPGAP45loxP–YFP–HX*	diCre Δku80
**5’UTR MyoB/C fw**	CGGGTACCGCTTCACTGGTTTATCGTGTCACGG	*Myosin B/C KO–Bleo*	loxPMyoA
**5’UTR MyoB/C rv**	GCAAGCTTGAAGTCACAGCCTCTCTCGGAAAACG	*Myosin B/C KO–Bleo*	loxPMyoA
**3’UTR MyoB/C fw**	CCTTAATTAATGCCTTTAAAGTGGACAAGGGTGAATGGACGG	*Myosin B/C KO–Bleo*	loxPMyoA
**3’UTR MyoB/C rv**	GGACTAGTTTGGTTCACGCAAGCGGTGTGTCG	*Myosin B/C KO–Bleo*	loxPMyoA
**YFP rv**	ATGGGCACCACCCCGG		
**HX fw2**	GCTACGACTTCAACGAGATGTTCCGCG		
**MLC1 3UTR rv2**	GCGAGAGCAAGAGTAGAGAAGTCGTCACC		
**MLC1 5′UTR fw2**	CCACACAATGGCTAGACTTGCTGCCC		
**GAP45 5′UTR fw2**	GCACACCGTGAATCTGCTGGT		
**GAP45 3'UTR rv2**	GCCAACTCGTGTGGAACAGACTGTCG		
**MyoB/C gene fw**	TACTTCCAATCCAATTTAATGCCTCCCGCAGTCCGTCAGACGCAATAGATGTTCG		
**MyoB/C gene rv**	TCCTCCACTTCCAATTTTAGCCGGTCTATCCGGCGCACAGGCCTCGAAGC		
**5’UTR MyoB/C fw**	GCTATCTCAACGGACAACGACTGCG		
**5’UTR MyoB/C rv**	CGTTGTCTCAGACTTTGC		
**3’UTR MyoB/C fw**	GCTGCACCACTTCATTATTTCTTCTG		
**3’UTR MyoB/C rv**	GCCAGAACCCAAAACTCTCCACTCAGACG		
