# Supplementary material for: The Toxoplasma Acto-MyoA Motor Complex Is Important but Not Essential for Gliding Motility and Host Cell Invasion
Source: PLoS One. 2014 Mar 14;9(3):e91819. doi: 10.1371/journal.pone.0091819 (PMC3954763; doi:10.1371/journal.pone.0091819)
Supplement: File S1 — (DOC) [file pone.0091819.s011.doc]

**Supporting Information**

**Estimates of the physical feasibility of the gelation-solation osmotic engine.**

Simple estimates demonstrate that the hypothesized gelation-solation osmotic engine is physically feasible. The protrusion force can be roughly estimated with Van't Hoff formula [1]: osmotic pressure at the leading edge P ~ cRT, where c is the molar concentration of the mobile cations, R is the gas constant and T is the absolute temperature. Assuming that c is in the order of ten micromolar (characteristic concentration of actin monomers, oligomers, short filaments and their counterions), this formula gives P ~ 10 – 100 pN/mμ^2^, which is lower but comparable with the protrusive force at leading edge of migrating cells [2]. Gel elastic modulus originating from ion-mediated electrostatic interactions and entanglement of semi-stiff polymers [3,4] can be on the order of 100 pN/mμ^2^ and thus withstand such pressure; upon partial disassembly such gel relaxes [5] and deforms (by up to microns) enough to cause the gel swelling. The Darcy permeability of the gel to the cytoplasmic flow [6], K ~ 0.01 mμ4/(pN×s) is high enough to allow the flow rate V ~ K×P/L ~ 1 mμ/s (L ~ 1 mμ is the characteristic length scale at the leading edge) that will not limit the pathogen propulsion.

1. Jiang H, Sun SX (2013) Cellular pressure and volume regulation and implications for cell mechanics. Biophys J 105: 609-619.

2. Heinemann F, Doschke H, Radmacher M (2011) Keratocyte lamellipodial protrusion is characterized by a concave force-velocity relation. Biophys J 100: 1420-1427.

3. Yao NY, Broedersz CP, Lin YC, Kasza KE, Mackintosh FC, et al. (2010) Elasticity in ionically cross-linked neurofilament networks. Biophys J 98: 2147-2153.

4. Koenderink GH, Atakhorrami M, MacKintosh FC, Schmidt CF (2006) High-frequency stress relaxation in semiflexible polymer solutions and networks. Phys Rev Lett 96: 138307.

5. Broedersz CP, Depken M, Yao NY, Pollak MR, Weitz DA, et al. (2010) Cross-link-governed dynamics of biopolymer networks. Phys Rev Lett 105: 238101.

6. Keren K, Yam PT, Kinkhabwala A, Mogilner A, Theriot JA (2009) Intracellular fluid flow in rapidly moving cells. Nat Cell Biol 11: 1219-1224.
